# Calcitonin Gene–Related Peptide Monoclonal Antibody Versus Botulinum Toxin for the Preventive Treatment of Chronic Migraine: Evidence From Indirect Treatment Comparison

**DOI:** 10.3389/fphar.2021.631204

**Published:** 2021-05-03

**Authors:** Jiajie Lu, Quanquan Zhang, Xiaoning Guo, Wei Liu, Chunyang Xu, Xiaowei Hu, Jianqiang Ni, Haifeng Lu, Hongru Zhao

**Affiliations:** ^1^ Department of Neurology, The First Affiliated Hospital of Soochow University, Suzhou, China; ^2^ Department of Neurology, Traditional Chinese Medicine Hospital of Kunshan, Kunshan, China

**Keywords:** chronic migraine, CGRP monoclonal antibody, botulinum toxin, indirect treatment comparison, meta-analysis

## Abstract

**Background:** The previously approved botulinum toxin and nowadays promising calcitonin gene-related peptide (CGRP) monoclonal antibody have shown efficacy for preventing chronic migraine (CM). However, there is no direct evidence for their relative effectiveness and safety. In this study, we conducted an indirect treatment comparison to compare the efficacy and safety of CGRP monoclonal antibody with botulinum toxin for the preventive treatment of chronic migraine.

**Methods:** Up to August 31, 2020, we systematically searched PubMed, Embase, and Cochrane Library Central Register of Controlled Trials (Central). Weighted mean difference (WMD) and relative risk (RR) were used to evaluate clinical outcomes. Indirect treatment comparison (ITC) software was used to conduct indirect treatment comparison.

**Results:** Ten studies were pooled with 6,325 patients in our meta-analysis. Both botulinum toxin and CGRP monoclonal antibody demonstrated favorable efficacy in the change of migraine days, headache days, HIT-6 score, and 50% migraine responder rate compared with placebo. In indirect treatment comparison, CGRP monoclonal antibody was superior to botulinum toxin in the frequency of acute analgesics intake (WMD = −1.31, 95% CI: −3.394 to 0.774, *p* = 0.02113), the rate of treatment-related adverse events (AEs) (RR = 0.664, 95% CI: 0.469 to 0.939, *p* = 0.04047), and the rate of treatment-related serious adverse events (RR = 0.505, 95% CI: 0.005 to 46.98, *p* < 0.001).

**Conclusion:** For chronic migraine patients, CGRP monoclonal antibody was slightly better than botulinum toxin in terms of efficacy and safety. In the future, head-to-head trials would be better to evaluate the efficacy and safety between different medications in the prevention of chronic migraine.

## Introduction

Migraine is a common disabling neurological disease. In the Global Burden of Diseases, Injuries, and Risk Factors Study (GBD 2016 Neurology Collaborators, 2019), migraine ranked the second in the four largest contributors of neurological disability-adjusted life years (DALYs) (2019). Chronic migraine (CM) is a well-defined subtype of migraine that affects 1–2% of the general population and 8% of individuals with migraine (Buse et al., 2012). The description of chronic migraine in ICHD-2018 is headache occurring on 15 or more days/month for more than 3 months, which, on at least 8 days/month, has the features of migraine (Headache Classification Committee of the International Headache Society (IHS), 2018). Chronic migraine is associated with greater personal and social burden, more frequent comorbidities, and may be related to persistent and progressive brain abnormalities (Bigal et al., 2008). Many patients do not respond well to, or comply with, conventional preventive therapies (Agostoni and Barbanti, 2019).

Prior to 2018, the only preventive treatment of CM approved by the U.S. Food and Drug Administration (FDA) was botulinum toxin A, which may take more than a month to reach maximum efficacy (Dodick et al., 2010). It is believed that the injection of botulinum toxin in the cranial–facial–neck region innervated by the trigeminal nerve can inhibit the release of CGRP from peripheral noxious sensory neurons, and it counteracts the impact of highly sensitive trigeminal nerve on the nociceptive sensory input of secondary neurons in the brainstem, thereby preventing central sensitization, which is a key pathophysiological mechanism of CM (Aurora and Brin, 2017). Calcitonin gene–related peptide (CGRP), which is abundant in trigeminal ganglion neurons and involved in central sensitization, is the best validated biomarker for migraine to date. Recently, monoclonal antibodies that target the CGRP pathway of migraine pathogenesis have been specifically developed for the prophylactic treatment of chronic migraine (Wrobel Goldberg and Silberstein, 2015; Israel et al., 2018). These anti-CGRP/R monoclonal antibodies have demonstrated good efficacy and excellent tolerability in phase II and III clinical trials (Bigal et al., 2015; Silberstein et al., 2017; Tepper et al., 2017; Detke and Goadsby, 2018; Dodick et al., 2019; Lipton et al., 2020).

While data show that botulinum toxin and the anti-CGRP monoclonal antibodies share the best documentation for the prevention of CM, there is not much information that compares the effectiveness and safety between the two to discuss which is a more potent preventative. In this case, indirect treatment comparison (ITC) is used to assess the relative effectiveness of two treatments by using a common comparator (Bucher et al., 1997). Since clinical trials rarely compare all treatments of interest, the ITC methodology is beneficial to inform healthcare decision-making (Jansen et al., 2014). In this study, we conducted an indirect treatment comparison to compare the effectiveness and safety of two medications using placebo as a common comparator. We try to provide reference for clinicians and patients suffering from chronic migraine in making their clinical decisions, and open new and promising scenarios in CM management.

## Methods

### Search Strategy

We searched PubMed, Embase, and Cochrane Library database for randomized controlled trials (RCTs) from inception to August 2020 testing the efficacy and safety of CGRP monoclonal antibody or botulinum toxin in the prophylactic treatment of chronic migraine. We used the following search terms: chronic migraine, CGRP monoclonal antibody, botulinum toxin, and randomized controlled trial. Besides, we read the references from the reviews and RCTs to avoid omissions of any relevant studies.

### Inclusion and Exclusion Criteria

Inclusion criteria were as follows: study type was RCT, enrolled participants were diagnosed with chronic migraine according to the diagnostic criteria of the International Headache Society, the study was to assess the efficacy and safety of CGRP monoclonal antibody or botulinum toxin in chronic migraine prophylaxis, the control group was placebo, and the study provided any available information on at least one targeted outcome measurement (migraine frequency, migraine days, responder rate, headache intensity, and adverse events).

RCTs with any of the following conditions were excluded: case reports, case reviews, *post hoc* analysis studies, retrospective studies, and cohort studies; providing insufficient data for meta-analysis; and the control group was not placebo. When multiple publications of the same RCT were found, we selected the article that reported the largest number of participants.

### Data Extraction

Literature information, inclusion and exclusion criteria, baseline characteristics of the object of the study, content of the expose or interfere, and outcome data were extracted from each study. The efficacy outcomes included changes in migraine days, headache days, HIT-6 score; changes in frequency of acute analgesics intake, and 50% migraine responder rate. Safety endpoints included incidence of treatment-emergent adverse events (AEs) and treatment-related serious adverse events (AEs).

### Statistical Analysis

**FIGURE 1:**
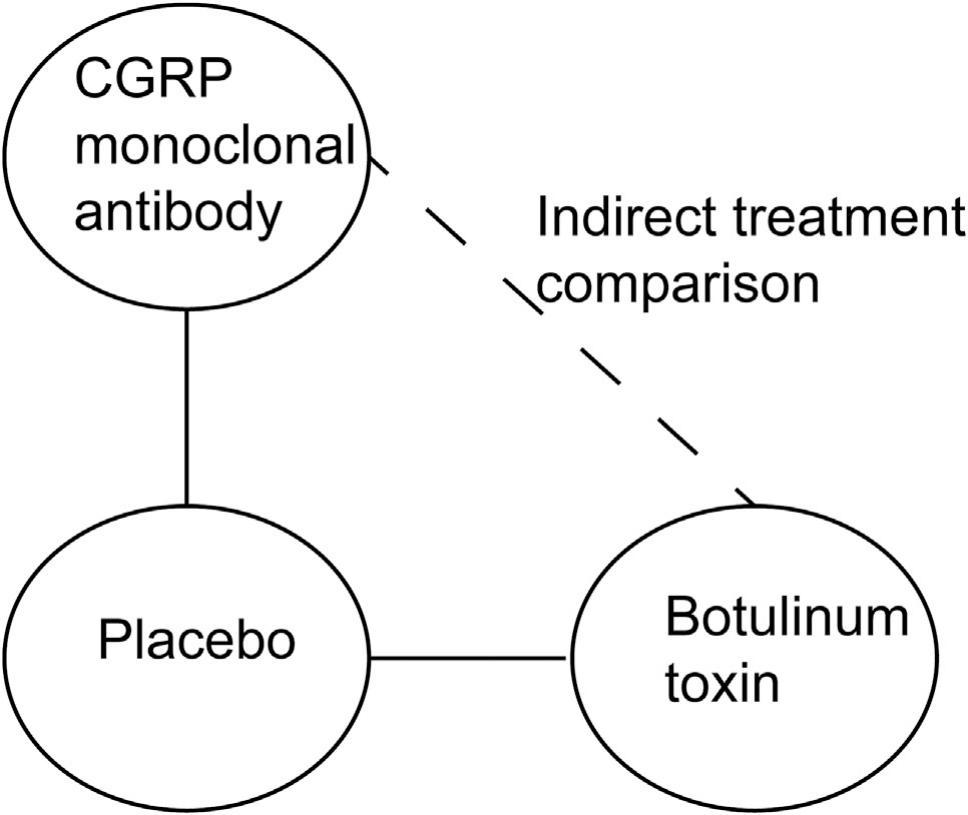
Standard pairwise Bucher method used to estimate the relative efficacy and safety of CGRP monoclonal antibody vs. botulinum toxin.

We summarized the characteristics of the included RCTs (number of studies and participants, details of interventions, outcome measures, major outcomes, and adverse events). Prior to the indirect treatment comparison, weighted mean differences (WMD) and relative risk (RR) with their 95% confidence intervals (CIs) were used to relatively evaluate the effect of CGRP monoclonal antibody or botulinum toxin on chronic migraine, and *I* ^2^ statistics were used to assess the statistical heterogeneity in direct comparisons. ITCs are performed using the standard pairwise Bucher method (Bucher et al., 1997). This method compares the magnitude of the treatment effect in each trial by assessing the difference between the treatment group and the placebo group (Figure 1). Review Manager 5.4 (Nordic Cochrane Center, the Cochrane Collaboration, Copenhagen, Denmark) and ITC 2.0 (Canadian Agency for Drugs and Technologies in Health, Ottawa, ON, Canada) were used to perform the analysis.

### Risk of Bias

Review Manager 5.4 was used to create the risk-of-bias plot in individual studies. To assess the risk of bias of RCTs, we adopted the unified standards of the Cochrane Collaboration, including selection bias, performance bias, detection bias, attrition bias, reporting bias, and other biases.

## Results

### Search Results and Baseline Characteristics

**FIGURE 2:**
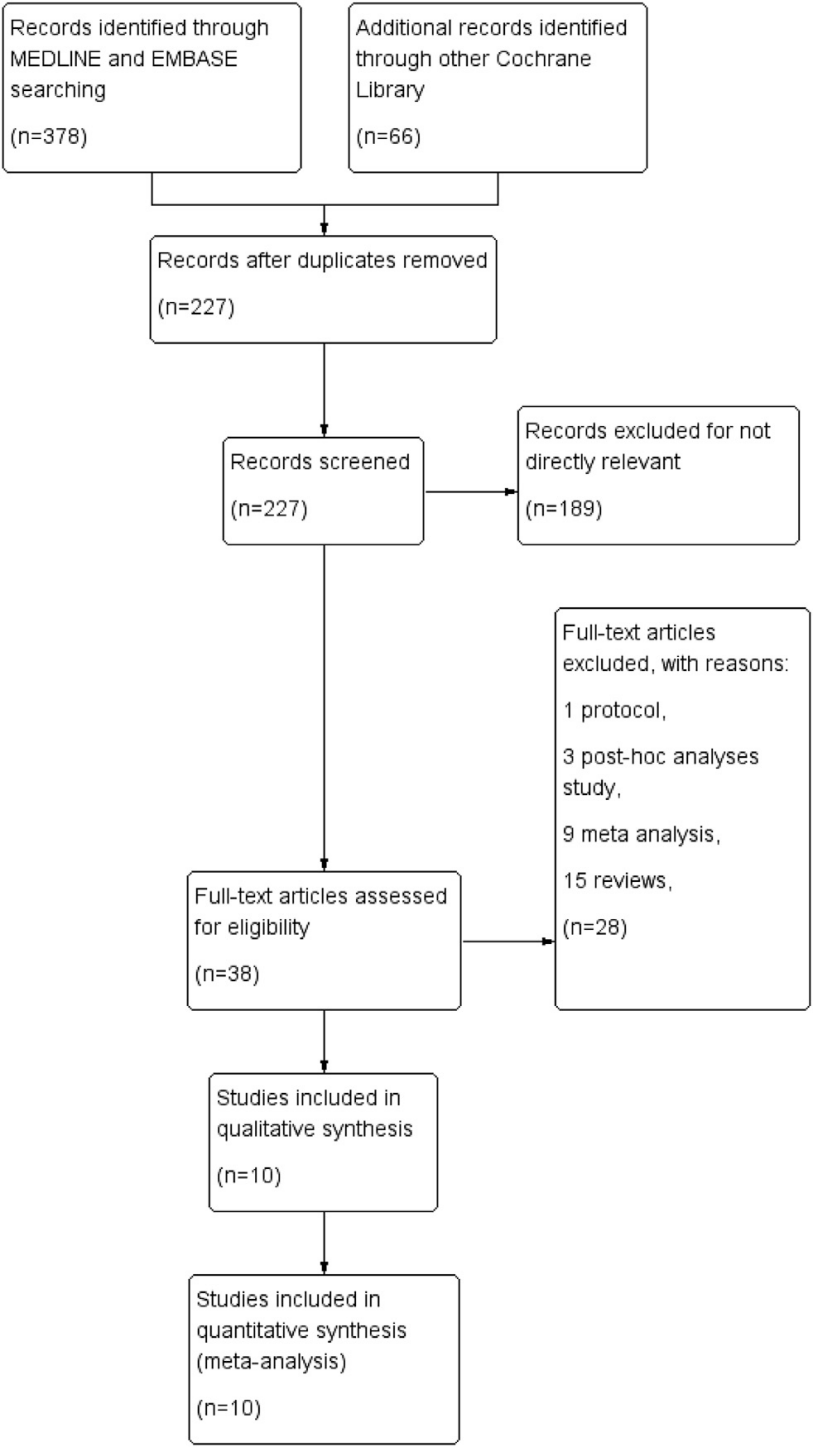
PRISMA flowchart of the study search, selection, and inclusion process.

We systematically searched for articles published before September 1, 2020 and found 444 articles related to this topic. 217 studies were removed due to duplicates. 189 studies were removed because they were not directly relevant to our study. Among the remaining reports, 10 studies containing 6,325 patients were included in our meta-analysis (Freitag et al., 2008; Aurora et al., 2010; Diener et al., 2010; Sandrini et al., 2011; Bigal et al., 2015; Silberstein et al., 2017; Tepper et al., 2017; Detke and Goadsby, 2018; Dodick et al., 2019; Lipton et al., 2020). An overview of the specific study selection process is plotted in Figure 2. Details of the baseline characteristics in each study are presented in Table 1.

**TABLE 1:** Characteristics of the included studies.

Trials phases Publication centre	Inclusion criteria	Exclusion criteria	Intervention Time period of assessment
Bigal; 2015 (NCT02021773) Bigal et al. (2015) phase 2b lancet neurology multicenter	Aged 18–65 years. A diagnosis of chronic migraine as defined by IHS ICHD-3β	Received onabotulinumtoxin a prior to 6 months before study entry, used opioids or barbiturate compounds for more than 4 days during the run-in phase, failed >2 medication categories or >3 preventive medications	Subcutaneous TEV-48125,675/225, 900 mg, or placebo in three 28-day cycles, 12 weeks
Detke; 2018 (NCT02614261) Detke and Goadsby, (2018) phase 3 neurology multicenter	Aged 18–65 years. A diagnosis of chronic migraine as defined by IHS ICHD-3β: migraine onset before 50 years of age	Persistent daily headache, cluster headache, head or neck trauma within the past 6 months, possible post traumatic headache, or primary headache other than CM, therapeutic antibodies during or within 1 year before the study	Monthly subcutaneous injections of placebo, galcanezumab 120 mg or 240 mg, 3 months
Dodick; 2019 (NCT02275117) Dodick et al. (2019) phase 2b cephalalgia multicenter	Aged 18–65 years. A diagnosis of chronic migraine as defined by IHS ICHD-3β: migraine onset at age 35 years and history of chronic migraine ≥1 year	Confounding pain syndromes, received botulinum toxin for migraine or for any other medical/cosmetic reasons within 4 months prior to screening	A single IV infusion of eptinezumab 300 mg, 100 mg, 30 mg, 10 mg, or placebo on day 0, 12 weeks
Lipton; 2020 (NCT02974153) Lipton et al. (2020) phase 3 neurology multicenter	Aged 18–65 years. A diagnosis of chronic migraine onset before 50 years of age and history of chronic migraine ≥1 year	Confounding pain syndromes, received botulinum toxin for migraine or for any other medical/cosmetic reasons within 4 months prior to screening	IV eptinezumab 100 mg, eptinezumab 300 mg, or placebo administered on day 0 and week 12, 12 weeks
Silberstein; 2017 (NCT02621931) Silberstein et al. (2017) phase 3 N ENGL J MDE multicenter	Aged 18–70 years. A diagnosis of chronic migraine for at least 12 months	Use of onabotulinumtoxinA during the 4 months before screening; the use of interventions or devices for migraine during the 2 months before screening; the use of opioid or barbiturate medications on more than 4 days during the preintervention period; lack of efficacy on at least two preventive medications	Abdominal subcutaneous fremanezumab quarterly, monthly, or placebo, 12 weeks
Tepper: 2017 (NCT02066415) Tepper et al. (2017) lancet neurology multicenter	Aged 18–70 years. A diagnosis of chronic migraine	Older than 50 years at migraine onset, had no therapeutic response with prophylaxis of more than three treatment, botulinum toxin injections in the head or neck region during the study and 4 months before the start of the baseline	Subcutaneous placebo, erenumab 70 mg, or 140 mg, every 4 weeks for 12 weeks, 3 months
Aurora: 2010 (NCT00156910) Aurora et al. (2010) cephalalgia multicenter	Aged 18–65 years migraine meeting the diagnostic criteria listed in ICHD-II, have ≥15 headache days with each day consisting of ≥4 h of continuous headache and with 50% of days being migraine or probable migraine	At increased risk exposed to onabotulinumtoxinA (e.g., neuromuscular diseases); other primary or secondary headache disorders; use of any headache prophylactic medication within 28 days before baseline	Injections every 12 weeks of onabotulinumtoxinA (155–195 U) or placebo, 24 weeks
Diener: 2010 (NCT00168428) Diener et al. (2010) cephalalgia multicenter	Aged 18–65 years migraine meeting the diagnostic criteria listed in ICHD-II, headache occurring on ≥15 days/4 weeks	At increased risk exposed to onabotulinumtoxinA (e.g., neuromuscular diseases); other primary or secondary headache disorders; use of any headache prophylactic medication within 28 days before baseline	Injections of onabotulinumtoxinA (155–195 U) or placebo every 12 weeks for two cycles, 24 weeks
Freitag: 2008 Freitag et al. (2008) headache diamond headache clinic	A 6-month history prior to baseline, of CM	Taken previous botulinum toxin of any serotype for any therapeutic reason, at increased risk exposed to botulinum toxin, a more painful condition than migraine pain	Botulinum toxin type a 100 units or placebo (sterile saline) in identical volumes was administered subcutaneously, 16 weeks
Sandrini: 2011 Sandrini et al. (2011) J headache pain multicenter	Aged 18–65 years fulfilling the diagnostic criteria for migraine without aura, plus medication-overuse headache with ≥15 headache days every 4 weeks in the past 3 months, with each headache day consisting of ≥4 h of continuous headache	Definite or suspected diagnosis of pathologies affecting neuromuscular function, including MG, Eaton–Lambert syndrome, and ALS, and presence of cervical pathologies or other factors liable to give rise to pericranial muscle disorders	16 intramuscular injections of onabotulinumtoxinA or placebo, 12 weeks

### Direct Comparison

#### Change in Migraine Days

**FIGURE 3:**
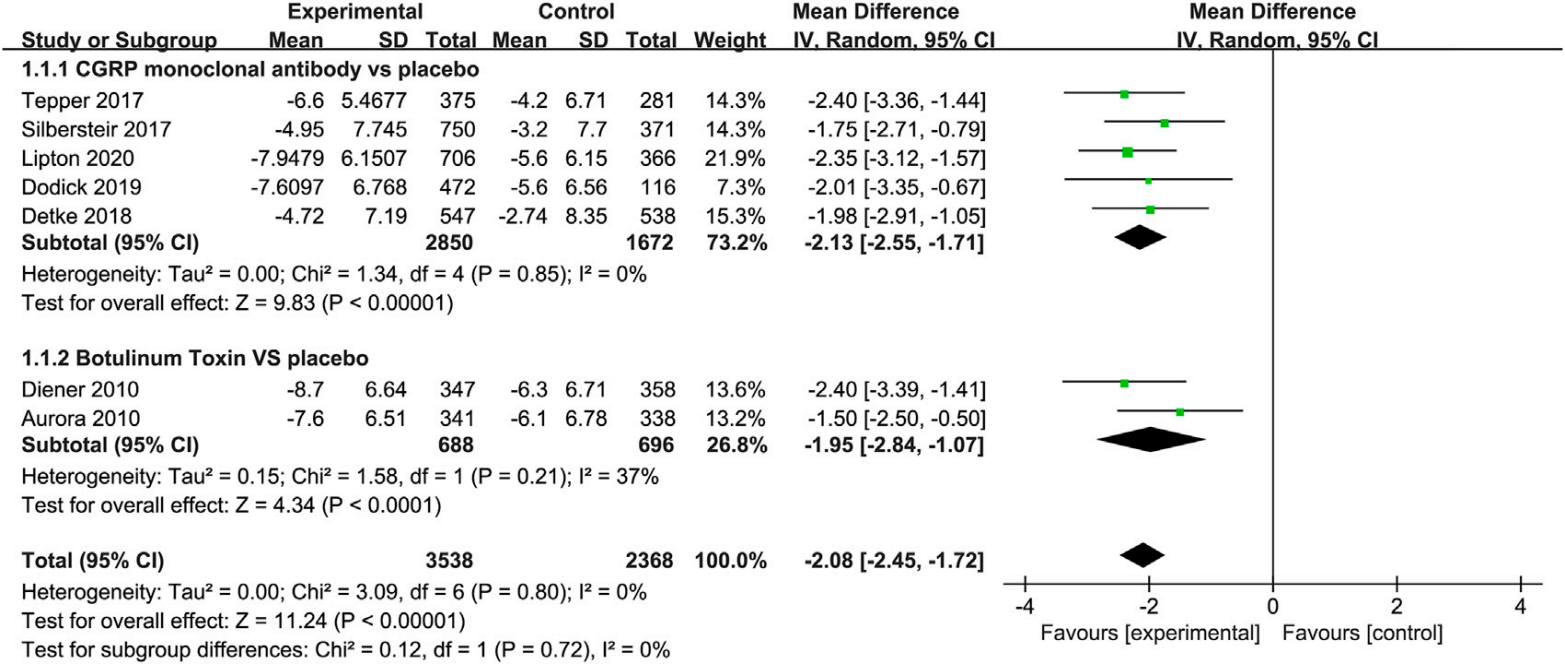
Pooled weighted mean differences of change in migraine days in the treatment group compared with placebo; diamond indicates the estimated relative risk with 95% confidence interval for the pooled patients.

Compared to placebo, the use of CGRP monoclonal antibody contributed to greater reductions in the number of monthly migraine days from baseline (WMD = −2.13, 95% CI: −2.55 to −1.71, *p* < 0.001), with low heterogeneity (*I* ^2^ = 0%). We also observed a significant difference in the botulinum toxin group compared to placebo (WMD = −1.95, 95% CI: −2.84 to −1.07, *p* < 0.001; Figure 3).

#### Change in Headache Days

**FIGURE 4:**
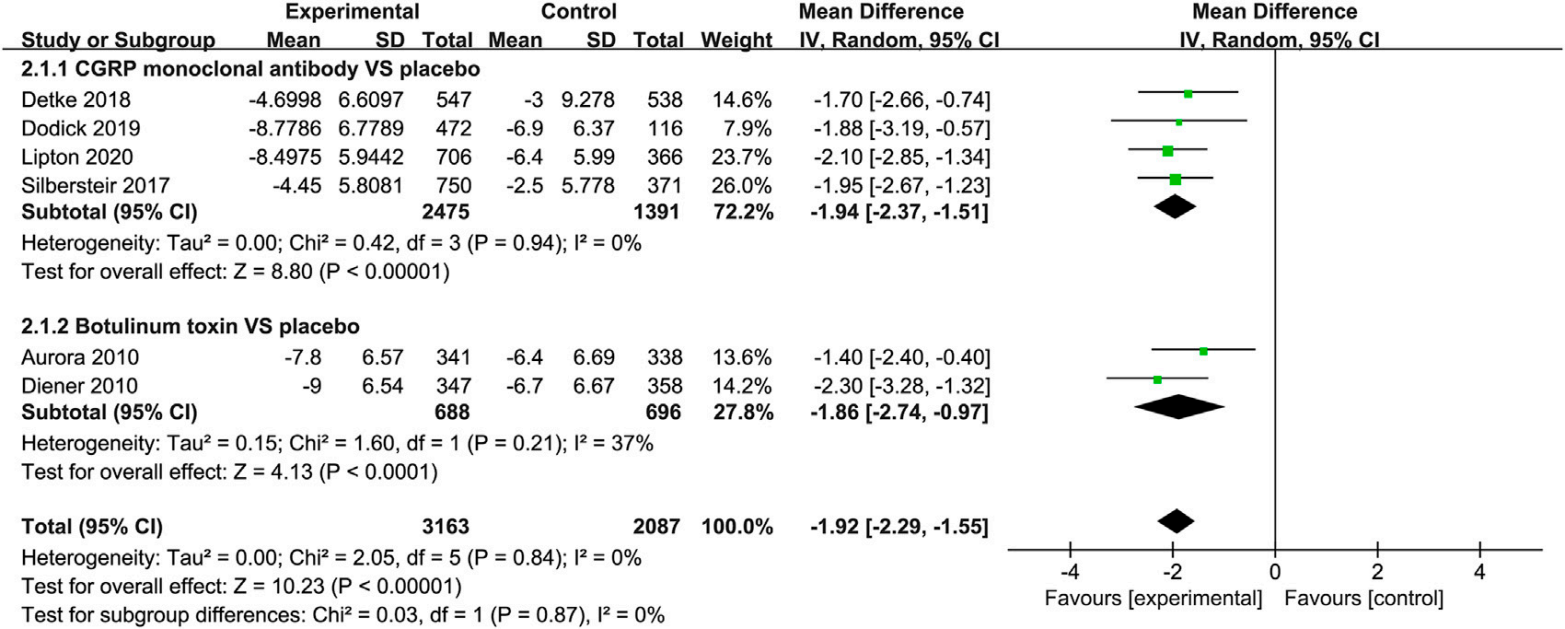
Pooled weighted mean differences of change in headache days in the treatment group compared with placebo; diamond indicates the estimated relative risk with 95% confidence interval for the pooled patients.

The studies listed in the meta-analysis showed that CGRP monoclonal antibody was superior to placebo in the mean change from baseline in headache days (WMD = −1.94, 95% CI: −2.37 to −1.51, *p* < 0.001). While in botulinum toxin vs. placebo, the change of headache days was also statistically significant (WMD = −1.86, 95% CI: −2.74 to −0.97, *p* < 0.001), with low heterogeneity (*I* ^2^ = 37%; Figure 4).

#### 50% Migraine Responder Rate

**FIGURE 5:**
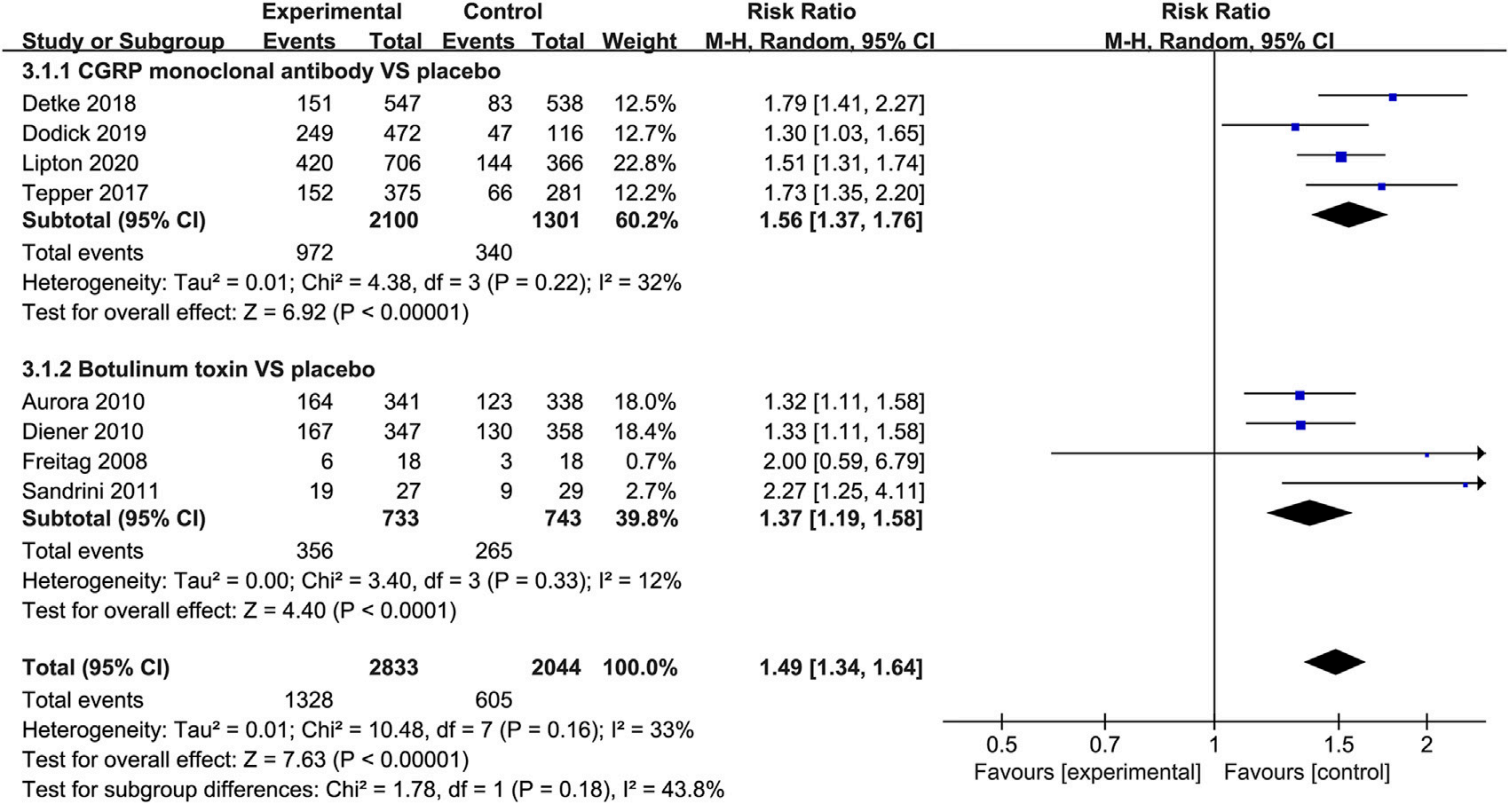
Pooled relative risk of the 50% migraine responder rate compared with placebo; diamond indicates the estimated relative risk with 95% confidence interval for the pooled patients.

When measured by the 50% migraine responder rate, CGRP monoclonal antibody was related to a higher response rate than the placebo group (RR = 1.56, 95% CI: 1.37 to 1.76, *p* < 0.001), with low heterogeneity (*I* ^2^ = 32%). The use of botulinum toxin was also associated with the increased 50% migraine response rate compared with placebo (RR = 1.37, 95% CI: 1.19–1.58, *p* < 0.001, *I* ^2^ = 12%; Figure 5).

#### Change in Frequency of Acute Analgesic Intake

**FIGURE 6:**
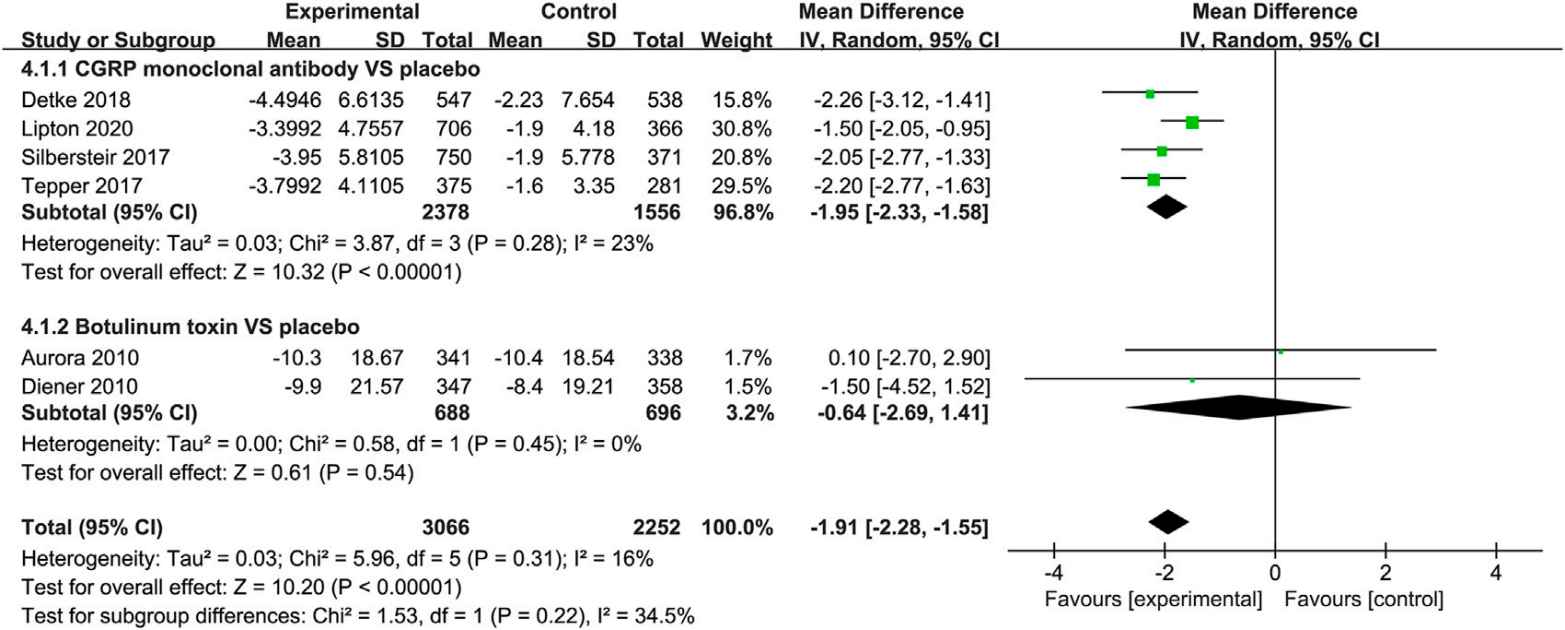
Pooled weighted mean differences of change in frequency of acute analgesics intake in the treatment group compared with placebo; diamond indicates the estimated relative risk with 95% confidence interval for the pooled patients.

The reduction in acute medication days in the CGRP monoclonal antibody group was greater than that in the placebo group (WMD = 1.95, 95% CI = −2.33 to −1.58, *p* < 0.001, *I* ^2^ = 0%). In the botulinum toxin vs. placebo trials, the difference between the two groups was small (WMD = −0.64, 95% CI: −2.69 to 1.41, *p* = 0.54). The botulinum toxin group did not relate to less acute analgesic intake (Figure 6).

#### Change in the HIT-6 Score

**FIGURE 7:**
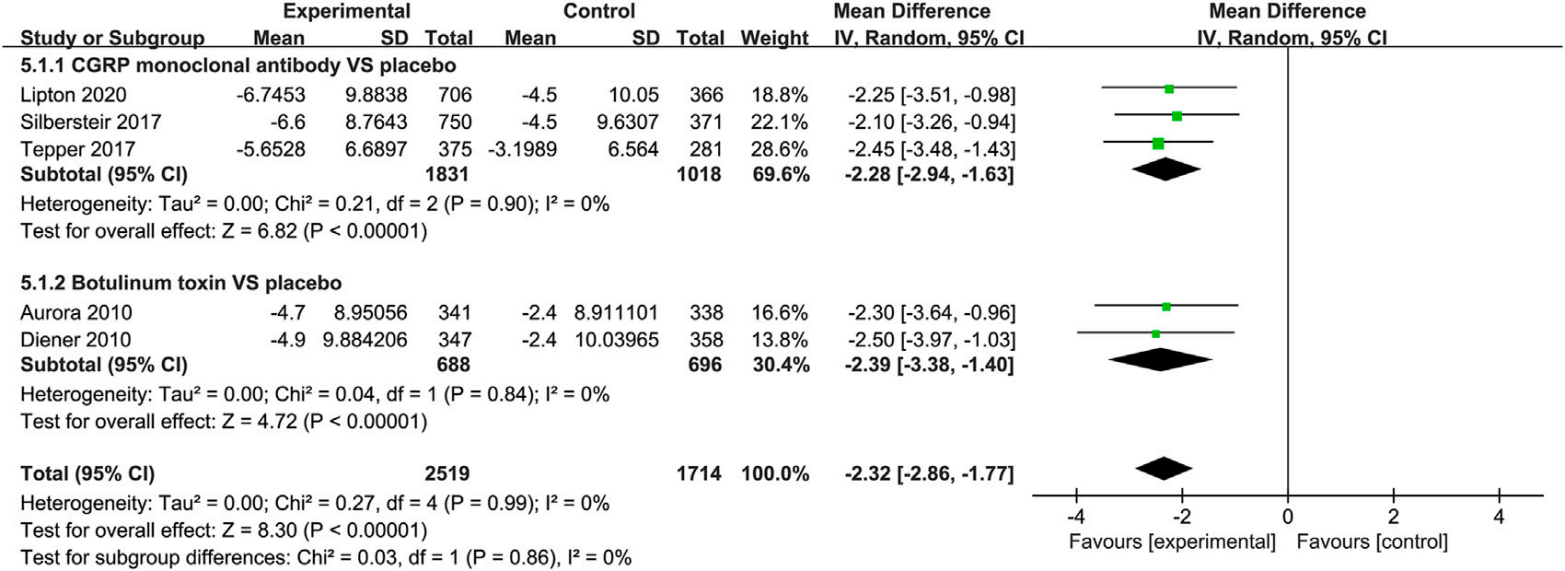
Pooled weighted mean differences of change in HIT-6 score in the treatment group compared with placebo; diamond indicates the estimated relative risk with 95% confidence interval for the pooled patients.

Both the CGRP monoclonal antibody group and the botulinum toxin group showed reduction in Headache Impact Test 6 (HIT-6) score (CGRP monoclonal antibody vs. placebo: WMD = −2.28, 95% CI: −2.94 to −1.63, *p* < 0.001, *I* ^2^ = 0%; botulinum toxin vs. placebo: WMD = −2.39, 95% CI: −3.38 to −1.4, *p* < 0.001, *I* ^2^ = 0%; Figure 7).

#### Safety Outcomes

**FIGURE 8:**
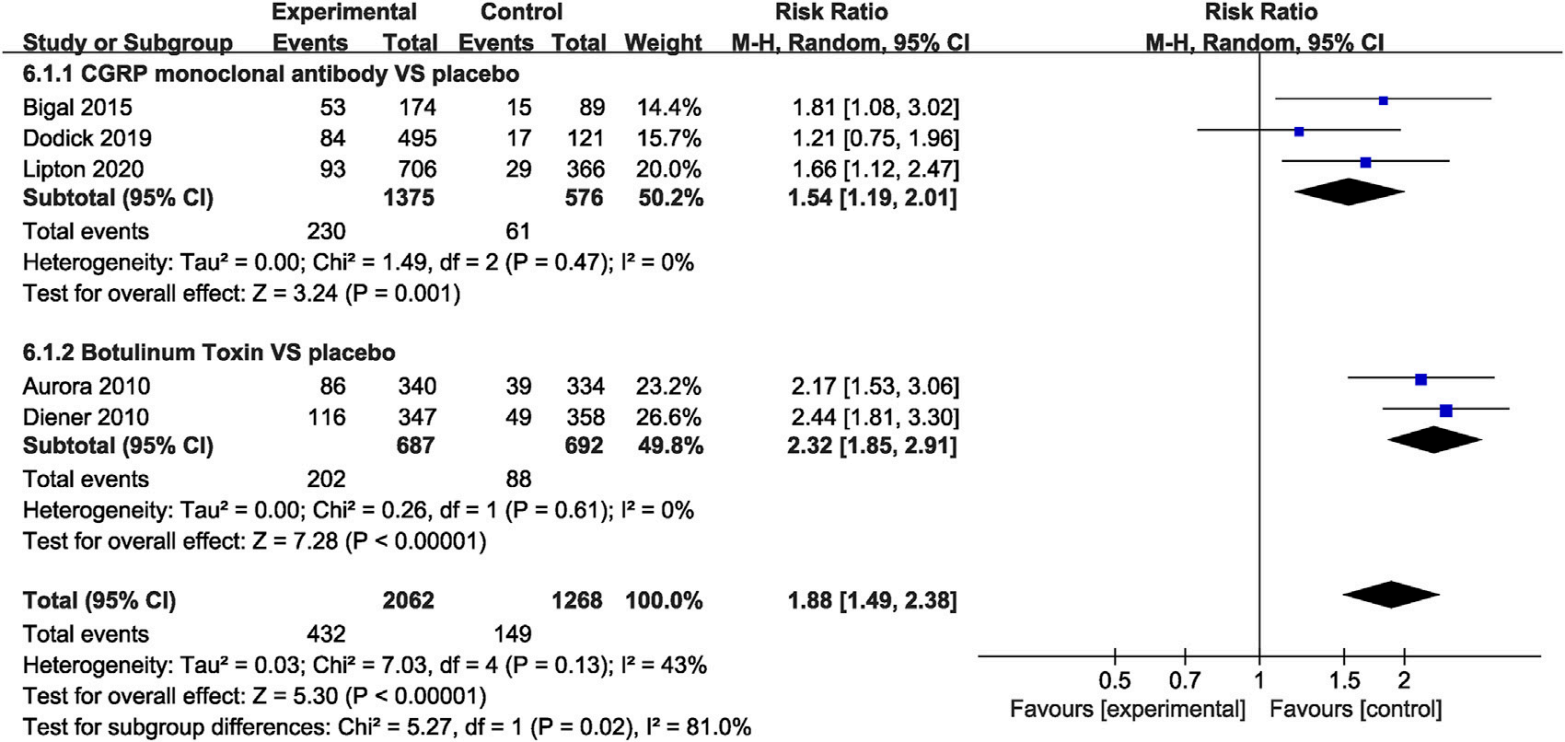
Comparison of treatment-related AEs in CGRP monoclonal antibody vs. placebo and botulinum toxin vs. placebo.

**FIGURE 9:**
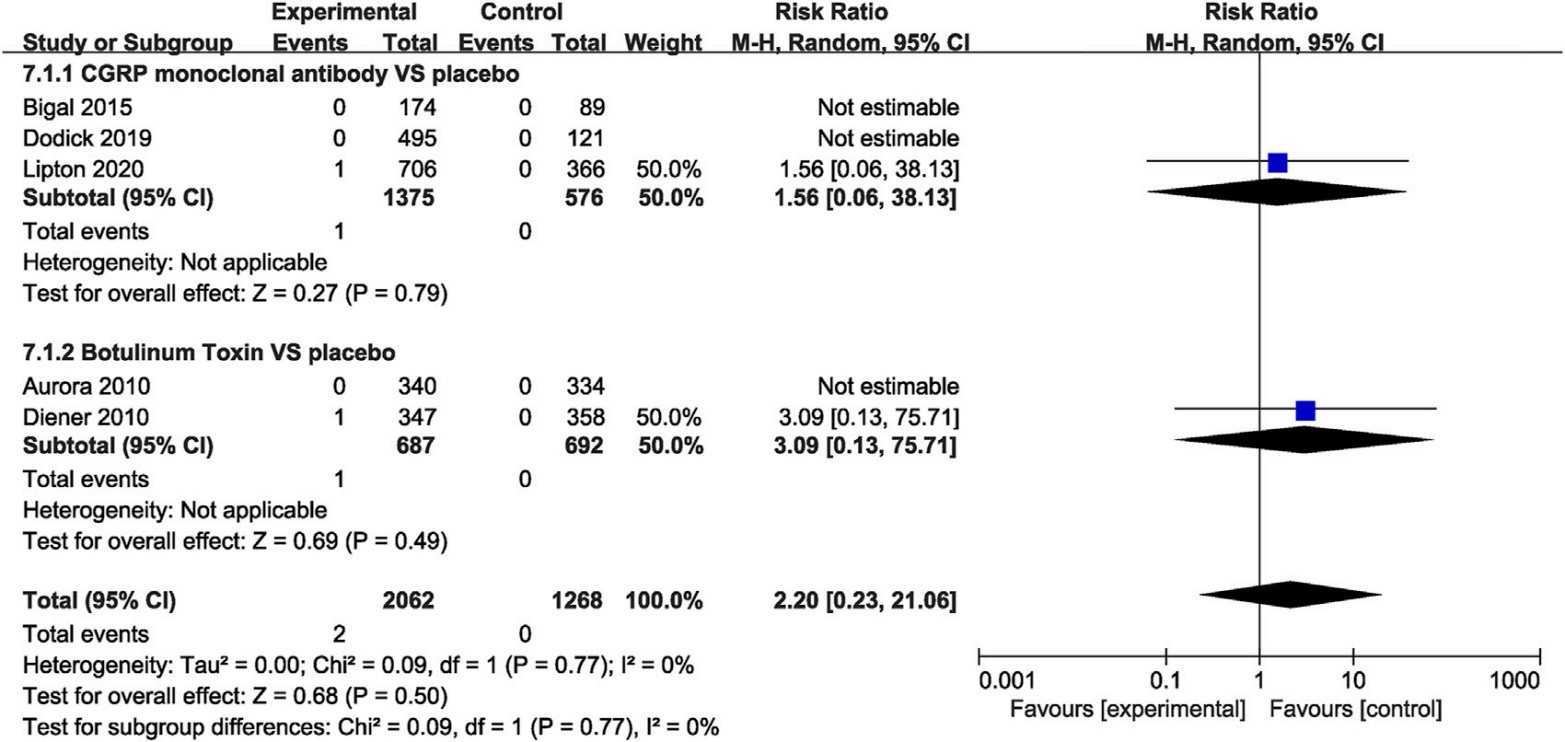
Pooled treatment–related serious AEs’ relative risk in CGRP monoclonal antibody and botulinum toxin.

In the CGRP monoclonal antibody group, a higher frequency of treatment-related AEs was identified than that in the placebo group (RR = 1.54, 95% CI: 1.19 to 2.01, *p* = 0.001). The botulinum toxin group was also associated with increased rates of treatment-related AEs compared with placebo (RR = 2.32, 95% CI: 1.85 to 2.91, *p* < 0.001; Figure 8). While both CGRP monoclonal antibody and botulinum toxin had no significant difference in treatment-related serious AEs compared with the placebo group (CGRP monoclonal antibody vs. placebo: RR = 1.56, 95% CI: 0.06 to 38.13, *p* = 0.79; botulinum toxin vs. placebo: RR = 3.09, 95% CI: 0.13 to 75.71, *p* = 0.49; Figure 9), no significant heterogeneity was found (*I* ^2^ = 0 for both outcomes).

### Indirect Treatment Comparison

The results of the ITC of CGRP monoclonal antibody and botulinum toxin are presented in Table 2. Compared to botulinum toxin, the CGRP monoclonal antibody group had more change in migraine days (WMD = −0.18), but the effect was not significant (95% CI: −1.16 to 0.8, *p* = 0.42530). No significant differences were found about the change of headache days (WMD = −0.08, 95% CI: −1.243 to 1.083, *p* = 0.72004) and 50% response rate (RR = 1.139, 95% CI: 0.942 to 1.376, *p* = 0.52496) between CGRP monoclonal antibody and botulinum toxin. While CGRP monoclonal antibody had a significant reduction in the frequency of acute analgesics intake (WMD = -1.31, 95% CI:-3.394 to 0.774, *p* = 0.02113). For the change of HIT-6 score, botulinum toxin seemed to drop more (WMD = 0.11), but the difference was not so significant (95% CI:-1.077 to 1.297, *p* = 0.61287). When it comes to safety outcome, the botulinum toxin group had a significant increase in the frequency of treatment-related AEs (RR = 0.664, 95% CI: 0.469 to 0.939, *p* = 0.04047) and treatment-related serious AEs (RR = 0.505, 95% CI: 0.005 to 46.98, *p* < 0.001).

**TABLE 2:** Indirect treatment comparison analysis of efficacy and safety outcomes of CGRP monoclonal antibody vs. botulinum toxin for the preventive treatment of chronic migraine.

Outcomes	Effective measure	95% CI	Test of association
Change in migraine days	−0.18	(−1.16 to 0.8)	0.42530
Change in headache days	−0.08	(−1.243 to 1.083)	0.72004
50% migraine responder rate	1.139	(0.942 to 1.376)	0.52496
Change in frequency of acute analgesic intake	−1.31	(−3.394,0.774)	0.02113
Change in the HIT-6 score	0.11	(−1.077,1.297)	0.61287
Incidence of treatment-related adverse events	0.664	(0.469,0.939)	0.04047
Incidence of treatment-related serious adverse events	0.505	(0.005.46.98)	0.00063

### Quality Assessment

**FIGURE 10:**
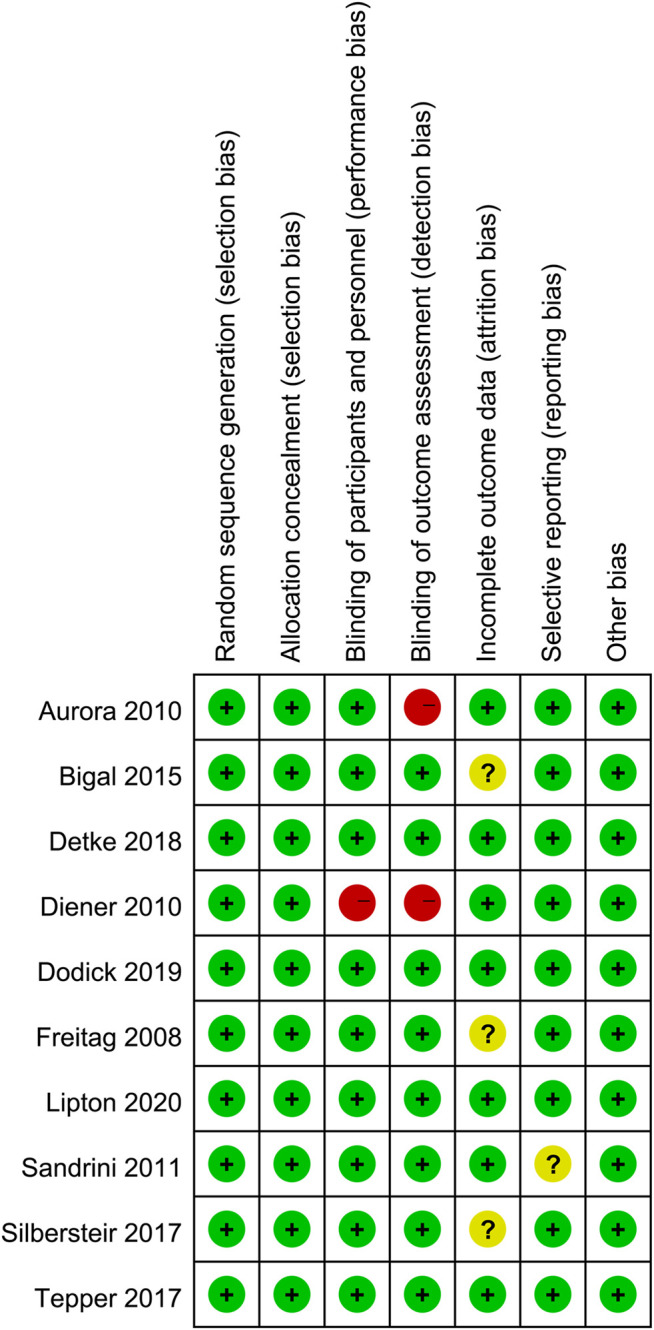
Summary table for potential bias analysis for included studies.

The independent risk of bias of the ten included studies is detailed in Figure 10. The bias risk of blind assessment of outcome data was high in the Aurora and Diener studies. Also, the Diener study had high performance bias. For incomplete outcome data, the Bigal, the Freitag, and the Silbersteir studies had unclear risk of bias. And the Sandrini study had an unclear risk of bias of selective reporting. In addition to these measures, other studies had low risks of bias.

## Discussion

To the best of our knowledge, this is the first meta-analysis followed by indirect treatment comparison to evaluate CGRP monoclonal antibody vs. botulinum toxin for the prevention treatment of chronic migraine in different outcome measures. In our meta-analysis, we pooled 6,325 patients from 10 randomized clinical studies, six studies compared CGRP monoclonal antibody with placebo and four studies compared botulinum toxin with placebo. It confirmed that both CGRP monoclonal antibody and botulinum toxin were effective in the prevention of CM. These results are in line with previous published RCT results. In the indirect comparison of treatment, there were no significant differences in changes of migraine days, headache days, HIT-6 score, and 50% migraine responder rate between CGRP monoclonal antibody and botulinum toxin. CGRP monoclonal antibody is superior to botulinum toxin in the frequency of acute analgesic intake and decreased rates of treatment-related AEs and treatment-related serious AEs. Overall, CGRP monoclonal antibody was slightly better than botulinum toxin in terms of efficacy and safety based on the evidence of this indirect meta-analysis.

Indirect comparisons make it possible to estimate effects in order to compare different interventions in systematic reviews even if there are no head-to-head trials of them. A meta-analysis using the Bucher method to assess the results of direct and indirect comparisons stated that in most cases (93%), results of adjusted indirect comparisons were not significantly different from those of direct comparisons (Song et al., 2003). However, the findings of indirect comparisons usually allow for less certainty in conclusions than the findings of appropriate pairwise meta-analyses of head-to-head trials, and the strength of inference from indirect comparisons is limited. The method may be susceptible to bias and may also produce overprecise answers. The internal validity and similarity of all trials involved should be carefully checked to investigate the underlying reasons for the discrepancies (Kiefer et al., 2015).

Botulinum toxin is, to date, the only approved treatment specifically for chronic rather than episodic migraine. A systematic review and meta-analysis revealed botulinum toxin A was associated with small to modest benefits in chronic daily headache and chronic migraine compared with placebo (Jackson et al., 2012). Subsequent comparison with standard preventive drugs showed that the efficacy of botulinum toxin A was similar to that of topiramate for the prevention of CM (Cady et al., 2011). Interestingly, botulinum toxin A treatment for CM significantly reduced symptoms of depression and anxiety, improved poor sleep quality and fatigue-related symptoms (Blumenfeld et al., 2019), and was also effective in chronic migraine patients with chronic medication overuse (Silberstein et al., 2013). However, in one RCT, in patients with chronic migraine and medication overuse, botulinum toxin did not afford any additional benefit over acute withdrawal alone (Pijpers et al., 2019). RCTs on the prevention of chronic migraine were mainly performed 10 years ago, and there have been few updates in recent 10 years.

Anti-CGRP antibodies are macromolecules that bind to CGRP ligands or their receptors, which can counteract the effects of excessive CGRP released from trigeminal sensory nerve fibers during migraine attacks (Tso and Goadsby, 2017). To date, the results from phase II trials and phase III trials of anti-CGRP/R monoclonal antibodies have been published on the efficacy and safety of prophylactic therapy in chronic migraine. The recent promising results with anti-CGRP monoclonal antibodies and previously approved botulinum toxin for the prophylactic treatment of CM prompt us to facilitate the use of evidence-based prophylactic treatment options. In the indirect treatment comparison, we found that the CGRP monoclonal antibody had more reduction in frequency of acute analgesic intakes, mainly attributing to long half-life, long duration of action, and long intervals between doses (Charles and Pozo-Rosich, 2019). They can be administered monthly (erenumab, fremanezumab, and galcanezumab) by subcutaneous injection or quarterly by subcutaneous (fremanezumab) or intravenous (eptinezumab) infusion. These agents may help to achieve rapid therapeutic effects within days to weeks and maintain longer (Pellesi et al., 2017). Also, botulinum toxin had less reduction in acute analgesics intake, which can be attributed in part to the fact that botulinum toxin takes effect relatively slowly and is generally effective over the long term. In the COMPEL study, treatment with botulinum toxin through week 108 provided continued improvement over 2 years (Blumenfeld et al., 2018). Other research suggested that continuing treatment with botulinum toxin for up to 12 months may be more beneficial to people with CM than early treatment withdrawal (Aurora et al., 2014). Among the four studies about botulinum toxin listed in our meta-analysis, the efficacy end points were assessed in week 24, week 16, and week 12. Follow-up periods of 3–6 months may not be sufficiently long to identify the clinical benefits of botulinum toxin compared with placebo, while CGRP monoclonal antibody has quick onset of action and may achieve rapid treatment effects over days to weeks. Schwedt found that erenumab showed early onset of efficacy within the first week of treatment in chronic migraine patients (Schwedt et al., 2018). CGRP monoclonal antibody had decreased rates of treatment-related AEs and treatment-related serious AEs. This may be explained by the method of administration. Botulinum toxin is administered to at least 31 injection sites across seven head and neck muscles (Tassorelli et al., 2018), while CGRP is more convenient and easier to operate using subcutaneous or intravenous infusion.

Nowadays, monotherapy is widely preferred in first-line prevention. Nevertheless, advancement in the understanding of migraine supports the development of rational combination regimens, potentially applicable in chronic migraine. A dual therapy may promote a synergistic effect through different pharmacological mechanisms (Pellesi et al., 2020). Most data show that CGRP monoclonal antibody inhibited Aδ- but not C-meningeal nociceptor responses to activation and sensitization by inflammatory mediators, whereas botulinum toxin prevented the activation of C- but not Aδ-fibers (Eftekhari et al., 2013; Burstein et al., 2014). Other research found that combined botulinum toxin and the CGRP antagonist blocked the activation and sensitization of high-threshold and wide-dynamic–range neurons, which was achieved through dual blockade of the Aδ and C classes of meningeal nociceptors (Melo-Carrillo et al., 2021). The combination of botulinum toxin and CGRP monoclonal antibody may be a more reasonable choice for the treatment of chronic migraine in the future, and the intriguing prospect should be substantiated through clinical trials evaluating efficacy, safety, and cost-effectiveness.

A limitation of our meta-analysis was that it was indirect comparison of treatment and the level of evidence was relatively lower than that of direct comparison. Between the studies on botulinum toxin and those on CGRP monoclonal antibody, the major difference in the patient’s election was presumably that refractory subgroup of CM patients only was largely excluded for the clinical trials about CGRP monoclonal antibody. We did not perform subgroup analysis in indirect comparison, such as medication overuse headache vs. non-medication overuse headache or refractory migraine vs. non-refractory migraine to fully explore the underlying differences between the two treatments. In addition, chronic migraine is a chronic problem, all the clinical trials cycles were relatively short, and it may not be long enough to prove the ultimate effectiveness of the treatment. Due to the lack of adequate clinical trial data, we did not choose a specific CGRP monoclonal antibody to compare with botulinum toxin. The injection way, dosage, and frequency of different CGRP molecules varied in trials, while botulinum toxin has a definite and univocal injection paradigm. It was administered intramuscularly to 31 sites across seven specific head/neck muscle areas. The discrepancy in methodology may partly influence the results. Moreover, unblinding might affect the efficacy of botulinum toxin. Study medication was injected at 31 sites including the forehead, which, compared with placebo, will eliminate wrinkling and possibly cause unblinding. In trials using similar designs, 85% of participants treated with botulinum toxin correctly guessed their treatment (Solomon, 2013). These limitations will largely be addressed in a head-to-head study, which will have a substantially longer duration, follow-up after the last dose, and analyses of different subgroup of CM.

## Conclusion

Both CGRP monoclonal antibody and botulinum toxin have shown good efficacy in preventing chronic migraine. CGRP monoclonal antibody was superior to botulinum toxin in the reduction of frequency of acute analgesics intake and lower incidence of treatment-related AEs and treatment-related serious AEs from our indirect comparison of treatment. In the future, head-to-head trials would be better to evaluate the efficacy and safety between different medications in the prevention of CM.

## Data Availability

The original contributions presented in the study are included in the article/Supplementary Material; further inquiries can be directed to the corresponding authors.

## Abbreviations

CGRP, calcitonin gene-related peptide; WMD, weighted mean difference; RR, relative risk; ITC, indirect treatment comparison; CM, chronic migraine; RCTs, randomized controlled trials; CIs, confidence intervals; AEs, adverse events; HIT-6, headache impact test 6
